# Long-term Monitoring Gait Analysis Using a Wearable Device in Daily Lives of Patients with Parkinson’s Disease: The Efficacy of Selegiline Hydrochloride for Gait Disturbance

**DOI:** 10.3389/fneur.2017.00542

**Published:** 2017-10-24

**Authors:** Mutsumi Iijima, Hiroshi Mitoma, Shinichiro Uchiyama, Kazuo Kitagawa

**Affiliations:** ^1^Department of Neurology, Tokyo Women’s Medical University School of Medicine, Tokyo, Japan; ^2^Tokyo Medical University, Medical Education Promotion Center, Tokyo, Japan

**Keywords:** Parkinson’s disease, gait disorder, gait analysis, selegiline, portable gait rhythmogram

## Abstract

**Objective:**

The aim of this study was to assess quantitatively the gait disorders in the daily lives of patients with Parkinson’s disease (PD) using with a newly developed portable gait rhythmogram (PGR), which has a trunk-mounted acceleration sensor and automatic gait-detection algorithm.

**Methods:**

Using the PGR, we recorded the daily walking profiles of 14 PD patients before and after the addition or increase in dose of an MAO-B inhibitor (selegiline, average dose: 4.0 mg/day) as part of their medicine regimen, and evaluated their gait using the unified Parkinson’s disease rating scale (UPDRS) and scores from a freezing of gait (FOG) questionnaire.

**Results:**

Before treatment with selegiline, the overall movements per 24 h was decreased below 0.41 m/s^2^ (mean − 1.5 SD) in eight patients. The mean gait acceleration was decreased below 1.94 m/s^2^ (mean − 2 SD) in 10 patients. The slope of the linear regression line was increased to 1.6 (mean + 1.5 SD) in eight patients. The cadence was increased to 124 steps/min (mean + 1.5 SD) in four patients. Based on continuous PGR recordings in the daily lives of the patients for 24 h, the addition or increase in dose of selegiline increased the amplitudes of gait accelerations in 4 of 10 patients (40.0%), widened the range of gait accelerations in 5 of 8 patients (62.5%), diminished the cadence in 4 of 4 patients (100%), and diminished the fluctuations in gait throughout the day in 12 of 14 patients (85.7%). The UPDRS III and FOG scores significantly improved after the addition or increase in dose of selegiline (*p* < 0.005, *p* < 0.01, respectively). However, changes in gait-related scores of UPDRS were not detected in six patients.

**Conclusion:**

Improvements in the gait fluctuations of PD patients after the addition or increase in dose of selegiline were detected using the PGR in the daily lives of the patients for 24 h. The PGR had a higher sensitivity for detecting the improvements than UPDRS scores.

## Introduction

Gait dysfunction is one of the cardinal symptoms of Parkinson’s disease (PD). Gait dysfunction interferes with the activities in the daily lives of PD patients and is resistant to dopamine replacement therapy in not only advanced but also early stages of the disease (1–6). In spite of the significance of gait dysfunction, it is difficult to assess gait deficits in PD patients quantitatively. The unified Parkinson’s disease rating scale (UPDRS) motor score is widely used to assess motor symptoms of PD patients (7). Since this score does not quantify tendencies in gait and posture improvement or deterioration, various kinds of gait analyses of PD patients have also been performed (6). However, these analyses do not necessarily elucidate gait deficits because of the following two reasons, as we previously reviewed (8).

First, gait analysis methods utilized to date do not measure elementary deficits in neural control mechanisms of gait. In gait analysis of short-distance walking, PD patients were described as exhibiting decreases in velocity and stride (1, 9–11), which, however, are commonly observed in patients with ataxic gait disorders (11). Walking with shortened stride, widened stance, slow speed, and prolonged double support period is the final common expression of frontal lobe dysfunction in the gait control system (12). Thus, disease-specific changes should be examined. Second, the short-distance walking in the laboratory could be improved by emotional stress effects. Taken together, the cycle and force during walking of PD patients should be measured over a long-term period in their daily lives to quantify their gait disorders.

We developed a new long-term monitoring device [portable gait rhythmogram (PGR)] that uses a trunk-mounted acceleration sensor and automatic gait detection algorithm. The PGR extracts gait induced-accelerations from other limb and trunk movements using a mathematical algorithm—the pattern matching method (13–16). This algorithm identifies the acceleration signals with high intensity, periodicity, and biphasicity as a possible gait sequence, from which gait peaks due to stride events are extracted by utilizing the cross-correlation and anisotropy properties of the signal. In our previous control experiments using metronome-guided walking, 11 healthy subjects and 12 PD patients were tested to evaluate the performance of the algorithm (15, 16). These studies showed that gait peaks were detected with an accuracy of more than 94% (15, 16). Thus, our new gait analysis system could detect gait-induced signals and define bradykinetic aspects in daily walking of PD patients.

The present study aimed to establish a gait analysis method for daily life that combines the modified detailed gait score with the newly developed PGR to assess the effects of anti-PD drugs quantitatively. The gaits of PD patients were examined before and after the addition or increase in dose of selegiline, a MAO-B inhibitor, as part of their medicine regimen. Selegiline was selected for the present study because the effects of this medication on gait disorders and motor fluctuations have been reported (17–22). Before and after the addition or increase in dose of selegiline, we quantified the degree of hypokinesia, the ability to produce sufficient forces for propelling the body during walking, the ability to scale the forces depending on the context during walking, and the fluctuations in these abilities throughout the day. We also examined whether the improvements in akinesia and gait parameters are homogeneous or heterogeneous.

## Materials and Methods

### Subjects

Using the PGR, we recorded the daily walking profiles of 14 PD patients (11 men and 3 women) with a mean age of 62.1 ± 14.9 years (±SD), including two patents reported (22). The profiles of patients are shown in Table 1. They represented all patients admitted to Tokyo Women’s Medical University Hospital between June 2010 and March 2013 who could walk unaided and showed no peak-dose dyskinesia during the “on” time. They included six patients with Hoehn and Yahr stage II and eight with stage III. The clinical status was examined using the UPDRS motor score "on" state (mean score of UPDRS Part III: 17.5 ± 9.0) (7). Clinically, 12 patients exhibited motor fluctuations. All patients were taking antiparkinsonian medication as follows: selegiline hydrochloride (2.5 mg/day, *n* = 3), levodopa (300–500 mg/day, *n* = 13), pramipexole (1.5–2.5 mg/day, *n* = 7), ropinirole (6 mg/day, *n* = 2), cabergoline (2 mg/day, *n* = 2), pergolide (750 μg/day, *n* = 1), amantadine (100–200 mg/day, *n* = 9), trihexyphenidyl (2.0–3.0 mg/day, *n* = 6), and zonisamide (100 mg/day, *n* = 1). l-DOPA equivalent doses were calculated for each patient (23), and the average was 591 mg. The study also included 17 age- and height-matched healthy controls (8 men and 9 women, age: 64.7 ± 4.5 years). Matching for age and height was based on the finding that gait cycle and floor reaction forces are influenced by these two parameters.

**Table 1:** Profile of patients with Parkinson’s disease.

Case	Age (years)	Sex	Duration of illness (years)	Hoehn and Yahr stage (on state)	UPDRS III (on state)	Motor fluctuation	l-DOPA (mg)	LED (mg)
1	75	M	6	3	23	Y	500	690
2	37	M	9	3	21	Y	200	700
3	67	F	20	3	23	Y	500	890
4	75	M	7	3	29	Y	300	650
5	75	F	8	3	40	Y	500	650
6	61	M	9	2	13	Y	400	665
7	58	M	6.5	2	15	N	300	500
8	48	M	6	3	19	Y	300	550
9	61	F	2.3	3	16	N	300	650
10	63	M	3.3	2	5	Y	200	225
11	72	M	4	3	15	Y	300	450
12	68	M	11	2	8	Y	400	633
13	46	M	12	2	8	Y	300	620
14	64	M	2	2	10	Y	0	400

*M, male; F, female; Y, yes; N, no; LED, L-dopa equivalent dose*.

Written informed consent was obtained from all subjects following a full explanation of this study. This study was conducted in accordance with the guidelines of the Committee of Medical Ethics of Tokyo Women’s Medical University (approval number 2439).

### Gait Measurements

#### Monitoring Acceleration

The PGR is a small device (size: 8 cm × 6 cm × 2 cm, weight: 80 g) that measures three dimensionally (*a_x_, a_y_, a_z_*) the accelerations accompanied by (1) limb and trunk movements and (2) accelerations induced by step-in and kick-off during gait (13–16). The PGR attaches to the waist of the patient, and records the above signals at a sampling rate of 10 ms. The data are automatically stored in a microSD card. When recording is completed, the absolute value of the acceleration vectors (*a*; $a^{2}=a_{x}^{2}+a_{y}^{2}+a_{z}^{2}$) is calculated and graphically displayed on a personal computer. A fully charged PGR can achieve 40 h of continuous recording.

#### Identification of Acceleration Induced by Gait Motion

The acceleration vectors related to stepping can be distinguished from those related to other limb and trunk movements or by unexpected artifacts, based on the mathematical method of pattern matching, as reported previously (14–16). The acceleration vectors related to stepping can be distinguished from those related to other movements, since the former have steep curves and appear rhythmically. Specifically, attention was focused first on a relatively strong signal region (e.g., *a* > 1 m/s^2^) in the acceleration time series, and a three-dimensional template wave (*a*_x_, *a*_y_, *a*_z_) with a duration of approximately 0.5 s was arbitrarily chosen around a local maximum point from that region. Then, the cross-correlation CC(*t*) was calculated between this wave and the whole time series at each time *t* using the following equation:

$$\mathrm{CC}(t)=\frac{\frac{1}{p}\sum_{i=1}^{p}\left[a_{x}(i)a_{x}(i+t)+a_{y}(i)a_{y}(i+t)+a_{z}(i)a_{z}(i+t)\right]}{{\left\{\frac{1}{p}\sum_{i=1}^{p}\left[a_{x}{\left(i\right)}^{2}+a_{y}{\left(i\right)}^{2}+a_{z}{\left(i\right)}^{2}\right]\right\}}^{\frac{1}{2}}\;{\left\{\frac{1}{p}\sum_{i=1}^{p}\left[a_{x}{\left(i+t\right)}^{2}+a_{y}{\left(i+t\right)}^{2}+a_{z}{\left(i+t\right)}^{2}\right]\right\}}^{\frac{1}{2}}}$$

where *p* is the length of the template wave. The obtained CC(*t*) is a scalar time series, showing pronounced rhythmic peaks even when the initial signal is too noisy to visualize periodicity with the naked eye. If the change in acceleration is caused by gait motion, the CC(*t*) peaks exhibit alternate changes in magnitude with time due to the left/right body sway during walking. Thus, we can detect correct peaks corresponding to one gait cycle. This pattern matching method also enabled us to distinguish the acceleration vectors caused by stepping from unexpected and large artifacts. Moreover, although the PGR moved during the recording period and the form of the acceleration vectors changed, we were able to identify the acceleration vectors continuously.

### Data Analysis

#### An Index of Hypokinesia

**Figure 1:**
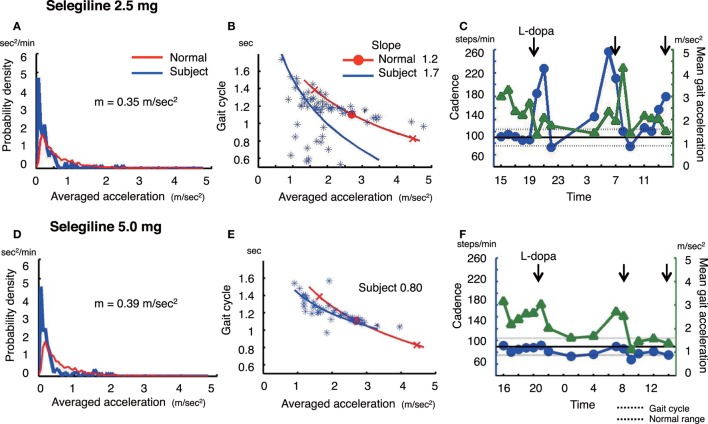
The summary of gait parameter changes in patient 1. Probability distribution of the averaged acceleration per 10 min, obtained from continuous recording for 24 h. All motion-induced accelerations are averaged per 10 min. Red lines: distribution curve for normal controls, blue line: distribution curve for a tested PD patient before **(A)** and after adding selegiline **(D)**. Relationship between gait acceleration and gait cadence. Both parameters were recorded continuously for more than 24 h and averaged every 10 min. Blue line: the regression line obtained from the all plots of the tested PD patient. Red line: the regression line obtained from the normal controls before **(B)** and after adding selegiline **(E)**. Serial changes in gait cadence and acceleration during daily activities. Left ordinate: the cadence (blue continuous line and circles), right ordinate: acceleration (green continuous line and triangles), abscissa: time, black dotted line: mean cadence ± 1 SD of normal subjects before **(C)** and after adding selegiline **(F)** (22).

**Figure 2:**
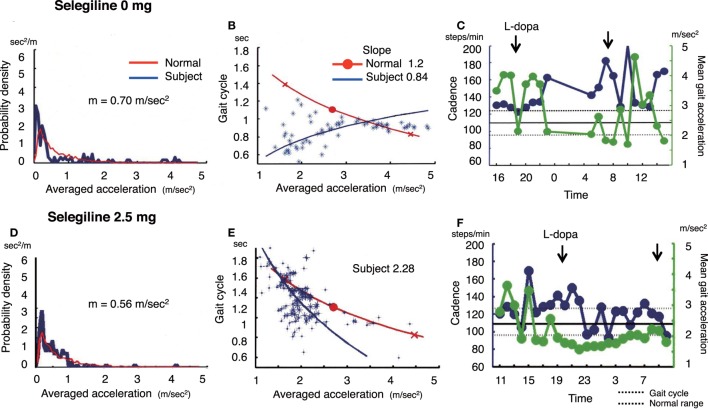
The summary of gait parameter changes in patient 2. Probability distribution of the averaged acceleration per 10 min, obtained from continuous recording for 24 h. All motion-induced accelerations are averaged per 10 min. Red lines: distribution curve for the normal controls, blue line: distribution curve for a tested PD patient before **(A)** and after adding selegiline **(D)**. Relationship between gait acceleration and gait cadence. Blue line: the regression line obtained from the all plots of the tested PD patient. Red line: the regression line obtained from the normal controls before **(B)** and after adding selegiline **(E)**. Serial changes in gait cadence and acceleration during daily activities. Left ordinate: the cadence (blue continuous line and circles), right ordinate: acceleration (green continuous line and triangles), abscissa: time, black dotted line: mean cadence ± 1 SD of normal subjects before **(C)** and after adding selegiline **(F)**.

**Figure 3:**
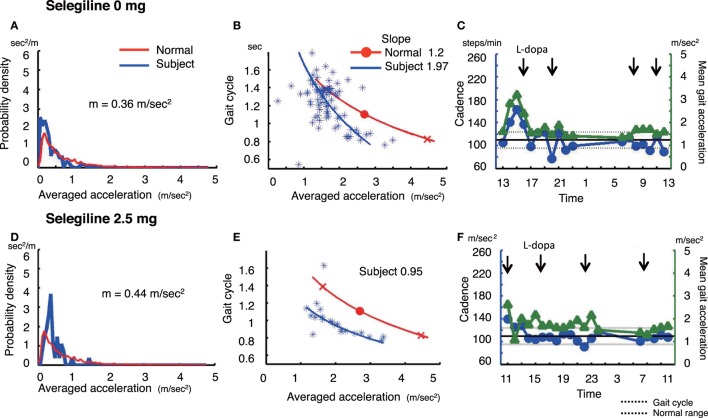
The summary of gait parameter changes in patient 3. Probability distribution of the averaged acceleration per 10 min, obtained from continuous recording for 24 h. Red lines: distribution curve for the normal controls, blue line: distribution curve for a tested PD patient before **(A)** and after adding selegiline **(D)**. Relationship between gait acceleration and gait cadence. Blue line: the regression line obtained from the all plots of the tested PD patient. Red line: the regression line obtained from the normal controls before **(B)** and after adding selegiline **(E)**. Serial changes in gait cadence and acceleration during daily activities. Left ordinate: the cadence (blue continuous line and circles), right ordinate: acceleration (green continuous line and triangles), abscissa: time, black dotted line: mean cadence ± 1 SD of normal subjects before **(C)** and after adding selegiline **(F)** (22).

We first analyzed the accelerations induced by all voluntary movements (all motion-induced accelerations), from the 24-h data recorded by the PGR. When the probability distribution of the 10-min averaged accelerations was calculated, the curve fit a gamma distribution. The mean was calculated and called the amount of overall movements per 24 h for each subject (8). The intersubject mean value of the amount of overall movements per 24 h was 0.69 ± 0.19 m/s^2^ in healthy controls (Figures 1A,D, 2A,D and 3A,D, red line).

#### Estimation of the Gait Cycle and Acceleration

Changes in gait cycle and gait acceleration amplitude were examined during the 24 h. The cycle and amplitude represent the mean values recorded every hour. Data were excluded when the step rate was <20/h (8, 13).

#### Estimation of Gait Acceleration-Cycle Plot

Since gait acceleration correlates with the floor reaction forces, we measured the gait acceleration as an index of floor reaction forces. The gait-related acceleration amplitudes and the duration of gait cycles were averaged for each 10-min recording and these averaged values were plotted. The averaged logarithmic values showed normal distribution, and the slope of the regression line was calculated using principal component analysis (8, 13–16). The regression line, obtained from the summed average of 17 healthy controls, is also shown (Figures 1B,E, 2B,E and 3B,E, red line).

### Evaluation of Motor Functions and Subjective Symptoms for Gait

Motor function was assessed before and 3 months after the addition or increase in dose of selegiline using part III of UPDRS. Gait severity was assessed using UPDRS item 29. This item classifies patients into four grades according to the severity of gait symptoms: (0) none, (1) rare freezing when walking; may have start hesitation, (2) occasional freezing when walking, (3) frequent freezing, occasionally falls from freezing, and (4) frequent falls from freezing. UPDRS was evaluated when patients were in the “on” state. Patients were asked to complete a freezing of gait (FOG) questionnaire (24) before and 3 months after the addition or increase in dose of selegiline.

### Statistical Analysis

Results were expressed as mean ± SD. The Student’s *t-*test was used for statistical analyses of the comparison of gait parameters between before and after the addition or increase in dose of selegiline. *p*-Values <0.05 were considered to indicate statistically significant differences.

## Results

All PD patients in the present study underwent 24-h measurements using the PGR, and the continuously recorded data were analyzed. Table 2 shows the UPDRS III scores, FOG scores, and PGR parameters for all patients before and after the addition or increase in dose of selegiline. Table 3 shows a summary of UPDRS III scores, FOG scores, and PGR parameters before and after the addition or increase in dose of selegiline. Before treatment with selegiline, the overall movements per 24-h was decreased below 0.41 m/s^2^ (mean − 1.5 SD) in eight patients. The mean gait acceleration was decreased below 1.94 m/s^2^ (mean − 2 SD) in 10 patients. The slope of the linear regression line was increased to 1.6 (mean + 1.5 SD) in eight patients. The cadence was increased to 124 steps/min (mean + 1.5 SD) in four patients. The UPDRS III and FOG scores significantly improved after the addition or increase in dose of selegiline (*p* < 0.005, *p* < 0.01, respectively). Twelve patients (85.7%) had improved UPDRS III scores, six (46.2%) showed improvements in item 29 of UPDRS, and seven (50.0%) had improved FOG scores. Regarding PGR parameters, improvements were seen in the amount of overall movements per 24 h in 5 (above 0.41 m/s^2^ or a 20% increase above the previous value) of 8 patients (62.5%), in the mean gait acceleration in 4 (20% increase above the previous value) of 10 patients (40.0%), and in the slope of the linear regression line in 5 (within mean + 1.5 SD) of 8 patients (62.5%). The cadence improved in 4 (within mean + 1.5 SD) of 4 patients (100%). The fluctuations in gait throughout the day diminished in 12 of 14 patients. These changes were not detected by UPDRS III gait-related scores in six patients.

**Table 2:** UPDRS III, FOG scores, and PGR parameters before and after adding selegiline.

Case	Selegiline (mg)		Total UPDRS III		UPDRS item 29		FOG score		
	Before	After	Before	After	Before	After	Before	After	
1	2.5	5	23	19	1	1	14	10	
2	0	2.5	21	15	2	1	13	13	
3	0	2.5	23	18	1	1	12	12	
4	0	5	29	14	1	0	2	2	
5	0	2.5	40	39	1	1	16	16	
6	0	5	13	11	1	1	9	6	
7	0	5	15	4	1	0	5	5	
8	0	5	19	9	1	0	11	7	
9	0	2.5	16	10	2	1	9	7	
10	2.5	5	5	5	0	0	2	1	
11	0	5	15	14	1	1	10	9	
12	0	2.5	8	4	1	0	8	8	
13	0	5	8	8	1	1	9	8	
14	2.5	5	10	7	1	1	9	7	

**Case**	**Amount of overall movement (m/s^2^)**		**Mean of gait acceleration (m/s^2^)**		**Slope of the linear regression line**		**Cadence (step/min)**		**Gait fluctuation**
	**Before**	**After**	**Before**	**After**	**Before**	**After**	**Before**	**After**	

1	0.35	0.39	1.82	1.94	1.7	0.8	120	95	Improved
2	0.7	0.56	2.86	1.95	0.84	2.28	152	100	Improved
3	0.36	0.44	1.68	2.04	1.97	0.88	96	125	Improved
4	0.31	0.37	1.11	1.66	1.61	1.37	112	116	Improved
5	0.38	0.44	1.08	1.12	3.12	2.57	164	86	Improved
6	0.42	0.37	1.9	1.66	1.71	1.15	130	106	Improved
7	0.42	0.34	2.05	2.18	1.44	1.08	99	107	Not improved
8	0.48	0.29	2.26	1.71	1.02	1.8	109	103	Improved
9	0.24	0.36	1.16	1.37	2.45	3.13	107	102	Improved
10	0.38	0.31	1.71	1.69	0.84	1.43	90	91	Improved
11	0.33	0.24	1.56	1.54	1.48	1.07	113	108	Improved
12	0.25	0.45	1.78	2.11	1.19	1.17	98	100	Not improved
13	0.44	0.56	2.14	2.83	2.27	1.62	146	106	Improved
14	0.44	0.52	1.58	1.78	2.24	2.44	105	99	Improved

**Table 3:** Comparison of UPDRS III, FOG scores, and gait components before and after adding selegiline.

	Before		After	*p*-Value	
Dosage of selegiline (mg)	0.4 ± 1.0		4.0 ± 1.4	<0.0001	

		**Abnormal patients (***n***)**			**Improved pateints (***n***) (abnormal patients: %)**

Total UPDRS part III score	18.1 ± 9.0	14	13.1 ± 10.0	<0.005	12 (85.7)
Item 29 (gait) score	1.1 ± 0.4	13	0.6 ± 0.4	0.03	6 (46.2)
FOG score	9.2 ± 4.9	14	7.9 ± 5.1	0.01	7 (50.0)
PGR parameter					
Amount of overall movement (m/s^2^)	0.39 ± 0.14	8	0.40 ± 0.01	0.7	5 (62.5)
Mean of gait acceleration (m/s^2^)	1.76 ± 0.1	10	1.83 ± 0.3	0.6	4 (40.0)
Slope of the linear regression line	1.70 ± 0.74	8	1.63 ± 0.74	0.6	5 (62.5)
Cadence (step/min)	117 ± 25.4	4	103 ± 14.3	0.07	4 (100)

*FOG, freezing of gait questionnaire, mean ± SD, n, number*.

In the following sections, we show some examples of the probability distribution of the average acceleration per 10 min, the gait acceleration-cycle plot, and cadence and gait acceleration during daily activities in two patients with increased selegiline dose (patient 1) and with newly added selegiline (patient 2 and patient 3).

### Patient 1

A 75-year-old man, who was diagnosed with PD 6 years ago, was being treated with 500 mg l-DOPA, 300 mg COMT inhibitor, and 2.5 mg selegiline. The amount of overall movements per 24 h was 0.35 m/s^2^ and extremely low compared with healthy controls (0.69 ± 0.19 m/s^2^) (Figure 1A). In the gait acceleration-cycle plot (Figure 1B), the regression curve for this patient was shifted to the left. The amplitude of the average acceleration (1.82 m/s^2^) was lower compared with healthy controls (2.78 ± 0.42 m/s^2^). Furthermore, the slope of the linear regression was steep, at 1.70, compared with healthy controls (healthy controls: 1.20 ± 0.29). When changes in the cadence (steps/min) and the gait acceleration amplitude were traced throughout the test day (Figure 1C), the patient showed an increase in the cadence >220 steps/min accompanied with a decrease in the gait acceleration amplitude at 19:00–21:00 and 05:00. Since a step cycle increase over 3 Hz is assumed to be FOG (25), the patient showed FOG at night, resulting in motor fluctuations. Three months after increasing the dose of selegiline to 5.0 mg, the amount of overall movements per 24 h remained almost unchanged (0.35 m/s^2^) (Figure 1D). In contrast, the regression curve in the gait acceleration-cycle plot (Figure 1E) was shifted to the right, almost within the range of the healthy controls. The average acceleration increased in amplitude (pre: 1.82 m/s^2^, post: 1.94 m/s^2^). The slope of the linear regression line flattened, decreasing from 1.70 to 0.8. Throughout the test day, the cadence was almost within the normal range, suggesting a disappearance of FOG (Figure 1F). Although UPDRS III changed from 23 to 19, the UPDRS III gait score remained unchanged (pre: 1, post: 1). By contrast, the patient’s FOG score improved from 14 to 10 points.

### Patient 2

A 37-year-old man who had been living with PD symptoms for 9 years, was taking 200 mg l-DOPA, 200 mg amantadine, and 3 mg pramipexole. He showed the normal amount of overall movements per 24 h (0.70 m/s^2^) (Figure 2A), and an average acceleration of 2.86 m/s^2^. In the gait acceleration-cycle plot (Figure 2B), the regression curve for this patient was shifted to the left. The slope of the linear regression line was steep, at 0.84. Figure 2C shows the fluctuations in cadence. The patient showed an increase in the cadence >160 steps/min accompanied with a decrease in the gait acceleration amplitude at 07:00–08:00, 10:00, and 14:00–15:00. After starting the patient on 2.5 mg selegiline, the slope of the linear regression line increased from 0.84 to 1.2 (Figure 2E). Throughout the test day, fluctuations in cadence improved, suggesting a disappearance of the fluctuations in gait (Figure 2F). Although the UPDRS III and UPDRS III gait scores changed from 21 to 15 and from 2 to 1, respectively, the FOG score did not change (pre: 13, post: 13).

### Patient 3

A 67-year-old woman who was suffered from the disease since 20 years ago, was taking 500 mg l-DOPA, 750 µg pergolide, 300 mg COMT inhibitor, and 2 mg trihexyphenidyl. She showed a decreased amount of overall movements per 24 h (0.36 m/s^2^) (Figure 3A). In the gait acceleration-cycle plot (Figure 3B), the regression curve for this patient was shifted to the left. Her average acceleration was low (1.68 m/s^2^), and the slope of the linear regression line was steep, at 1.97. Figure 3C shows the fluctuations in cadence. At 20:00 and 22:00 and 08:00, 10:00, and 12:00, the cadence slowed, suggesting that the patient walked with a bradykinetic movement. Due to her gait disorders and wearing off, 2.5 mg selegiline was added to her medicine regimen. After the addition of selegiline, the amount of overall movements per 24 h slightly increased from 0.36 to 0.44 m/s^2^ (Figure 3D). In the gait acceleration-cycle plot (Figure 3E), the regression curve was shifted slightly to the right, and the amplitude of the average acceleration increased (pre: 1.68 m/s^2^, post: 2.04 m/s^2^). However, the slope of the linear regression line flattened, decreasing from 1.97 to 0.95. Throughout the test day, the cadence was almost within the normal range, suggesting a disappearance of the fluctuations in gait (Figure 3F). Although the UPDRS III score changed from 23 to 18, the UPDRS III gait score remained unchanged (pre: 1, post: 1). No changes were detected in the modified gait score (pre: 12, post: 12).

## Discussion

### High Sensitivity of PGR for Detection of Drug-Induced Improvements in Motor Symptoms of PD Patients

Average UPDRS III, item 29 (gait), and FOG scores were significantly improved after the addition or increase in dose of selegiline; however, item 29 of UPDRS was improved in only six patients (46.2%). PGR quantitatively revealed changes in the gaits of the patients. Although the amount of overall movements per 24 h changed in 5 of 8 patients, the amplitude of the gait acceleration increased in 4 of 10 patients, the range of the gait acceleration correspondingly widened in 4 of 10 patients, and the slow cadence improved in 4 of 4 patients after increase in dose of selegiline. That is, the addition or increase in dose of selegiline (average dose: 4.0 mg/day) did not significantly improve the hypokinesia, but did increase the amplitude and range of forces some patients used when walking. Due to these improvements, the patients would be subjectively consciousness of improvements in their gait. In 12 of 14 patients, motor fluctuations disappeared. For example, FOG disappeared in the patient 1, and the duration of a slower cadence shortened in patient 2. These results show that the PGR had high sensitivity to detect anti-PD drug-induced changes in gait disorders.

Walking in daily life differs from that in short-distance walking examinations. Figure 1C shows the range of the cadence from 70 to 260 steps/min for walking in daily life. This variability in the cadence suggests that subjects change the mode of their gait depending on the circumstances. When a suitable mode of the gait is chosen, a particular set of the cycle and the amplitude for the gait is automatically determined, as shown by the regression line in the gait acceleration-cycle plot, (see Figures 1B,E, 2B,E and 3B,E). Although there are numerous combinations of the cycle and amplitude, it is interesting that a particular set of these parameters is determined as if they attract the neural controls. Thus, the goal of this method is to measure the average value and the range using the gait acceleration-cycle plot to examine the abilities of the patient to generate forces and the scaling of the amplitudes of these forces in the patient’s gait during daily life. These elemental capacities for gait cannot be detected in short-distance walking, and are clarified in context-dependent voluntary walking in daily life. The present results suggest that after the addition or increase in dose of selegiline to their medicine regimen, some patients were able to produce sufficient forces enough for propelling their body during walking and to scale their amplitudes depending on the circumstances when walking. Consequently, it was possible to define the physiological aspects of the gait disorders that were improved by the medication.

To minimize differences in daily activities depending on the test day, it is better to record continuously for 3 days. However, our previous report showed that significant differences were not observed in the average parameters, such as the amount of overall movements per 24 h and the mean gait acceleration, when the patients were asked to perform similar patterns of movement during their daily lives (26). Differences depending on the test day might be small if the mean and the range of these values are examined.

### Heterogeneous Improvement of Hypokinesia and Gait Bradykinesia

The present results also show that improvements in hypokinesia (a decrease in the amount of overall movements per 24 h) and gait disorders did not occur in parallel, suggesting that manifestation of movements and gait might be controlled independently. Moreover, the improvements in gait parameters were heterogeneous. When all patients were compared between before and after the addition or increase in dose of selegiline to their medicine regimen, significant differences were observed in cadence, not gait amplitude parameters (mean gait accelerations and slope of the regression line), which might also suggest heterogeneous improvement between rhythm control and force production. This assumption is supported by a heterogeneous impairment between rhythm generation mechanisms and force production mechanisms in the early stage of PD (26).

Walking movement is assumed to be manifested through activation of the midbrain locomotor region–reticulospinal tract system on the rhythm generators in the spinal cord (27). This neural circuit is controlled by dopaminergic and noradrenergic pathways. The dopaminergic nigra-striatum pathways facilitate the disinhibition in the basal ganglia, resulting in excitatory effects on the midbrain locomotor region to initiate walking as well as on the thalamo-cortical circuits to initiate voluntary limb movements (27). Furthermore, the noradrenergic pathways appear to facilitate rhythm generation in the spinal cord. Selegiline is reported to elicit two kinds of pharmacological effects, (1) an inhibition of MAO-B, which increases endogenous dopamine levels and (2) an increase in synthesis of phenylamine, which facilitates the release of noradrenaline (28). The present finding that selegiline improved differentially the gait disorder, but not the hypokinesia, suggests that selegiline might facilitate the noradrenergic pathways alone at a low dose (2.5–5.0 mg). Such a heterogeneous improvement in motor symptoms might occur when taking anti-PD drugs, which would be elucidated by monitoring simultaneously the index of hypokinesia in all movements and the physiological parameters in gait disorders.

The PGR was able to detect motor fluctuations and improvement during walking in daily life in a small number of PD patients. However, to confirm these results, a prospective cohort study with a larger number of patients is needed. Quantitative evaluations of gait by the PGR could be useful for evaluating motor function in patients with cognitive impairment who are unable to describe properly their symptoms in a patient diary or in patients who live alone and are unable to evaluate their motor symptoms themselves.

## Conclusion

We established a gait analysis method that combines long-term monitoring of movement during daily life using a newly developed PGR and gait scores to assess quantitatively the effects of selegiline in patients with PD. The PGR detected the improvement in fluctuations in gait by the addition or increase in dose of selegiline with a higher sensitivity than the gait scores. Evaluation of gait analysis by PGR is useful for quantitative gait evaluation and judgment of drug effects.

## Ethics Statement

Informed consent was obtained from all subjects. All procedures were conducted in accordance with the guidelines of the ethics committee of our institution.

## Author Contributions

Research project conception and research project organization: MI, HM, and SU. Research project execution, statistical analysis design, statistical analysis execution, and manuscript preparation and writing of the first draft: MI and HM. Statistical analysis review and critique: SU and KK.

## Conflict of Interest Statement

The authors declare that the research was conducted in the absence of any commercial or financial relationships that could be construed as a potential conflict of interest.
